# Implementation of training to improve communication with disabled children on the ward: A feasibility study

**DOI:** 10.1111/hex.13283

**Published:** 2021-05-28

**Authors:** Kath Wilkinson, Rebecca Gumm, Helen Hambly, Stuart Logan, Christopher Morris

**Affiliations:** ^1^ Peninsula Childhood Disability Research Unit (PenCRU) and National Institute of Health Research Applied Research Collaboration for the South West Peninsula (penARC), University of Exeter Medical School University of Exeter Exeter UK; ^2^ Sheffield Children's NHS Foundation Trust Sheffield UK

**Keywords:** communication, disabled children, implementation, inpatient, training

## Abstract

**Background:**

Parents of disabled children report poorer inpatient experiences when they stay in hospital, and some staff report finding communicating with disabled children challenging. This study tested the feasibility of implementing a training package for staff on paediatric wards to improve communication with disabled children, especially those with communication difficulties, and their families. The package was developed with parent carers and clinicians, and comprises a manual, a video of parent carers talking about real experiences, discussion points and local resources. The 50‐minutes training is intended for in‐house delivery by local facilitators.

**Methods:**

Thirteen training sessions were delivered in paediatric wards across four hospitals in England, totalling 123 staff who took part. Participants completed questionnaires before (n = 109) and after (n = 36) training, and a sample of champions (senior clinicians) and facilitators were interviewed at the end of the study.

**Results:**

Facilitators found the training easy to deliver, and participants felt they took away important messages to improve their practice. After the training, further changes were reported at an organizational level, including offering further training and reviewing practices.

**Conclusions:**

This study provides supporting evidence for the implementation of a low‐cost, minimal‐resource training package to support staff communication with children and their families in hospitals. It provides promising indication of impact on behavioural change at the individual and organizational level.

**Patient and public contribution:**

Parent carers identified the need and helped to develop the training, including featuring in the training video. They were also consulted throughout the study on research design, delivery and reporting.

## 1 BACKGROUND

Disabled children have the right to be treated with respect, involved in their care and consulted on decisions that affect them, as asserted by the UN Conventions on the Rights of the Child^1^ and the Rights of Persons with Disabilities.^2^ Unfortunately, poor experiences of care are still widely reported for disabled children. Parents and carers whose child was described by researchers as having a developmental disability, mental health condition, neurological condition or ‘other long‐term condition’ reported more negative experiences relating to ‘respect for their child's individual preferences and needs’ in the UK Care Quality Commission Survey in 2018, compared with the parents/carers of other children aged 0‐15 years, mirroring results in previous surveys.^3^

Recent work undertaken by Oulton and colleagues has identified inconsistent policies, systems and practices in English hospitals to support the care of children and young people with learning disabilities.^4^ Oulton also highlighted varying staff reports of the value of dedicated roles such as learning disability nurses.^5^ Staff report a reliance on parents to inform them about their child's needs,^6^ and parents describe a real need for a partnership with professionals in their child's care.^7^

Following Alderson's landmark study about children's participation in consent to surgery,^8^ there has been much research on children's involvement in outpatient consultations.^9^, ^10^ However, there has been relatively less research focusing on children's experience as inpatients, particularly that of disabled children. Our structured review of qualitative research on the experience of disabled children as inpatients suggested that communication mediates many aspects of their experience, but is often inadequate.^11^ Good communication can help to alleviate adverse emotional states and contribute to a more positive perception of the environment. Communication in hospital can be particularly challenging for children with learning disabilities^12^ and those who use communication aids.^13^ Parents of disabled children who struggle with communication often feel unable to leave their children because of concerns about communication.^14^, ^15^

Our own qualitative research confirms that hospital staff report finding communicating with disabled children on wards challenging,^11^ and other research has found staff expressing personal frustration with failing to meet children's communication needs.^4^, ^16^, ^17^ Staff identified several barriers to communication with disabled children, including the following: time pressures on a busy ward; low priority given to communication; a lack of information about an individual's communication needs; and a lack of experience. In contrast, making time, building a rapport with a child, previous experience of working with children and a family‐centred outlook have all been identified as positive influences.^11^, ^17^, ^18^

Parent carers who are part of the Peninsula Childhood Disability Research Unit (PenCRU) Family Faculty identified a need for something to be done to support hospital staff in order to improve their communication with disabled children and their subsequent experience as inpatients. Using intervention mapping, a training package was co‐developed in partnership with parent carers and health professionals (see our previous paper: 19). The training aims to support and empower staff to become more confident when communicating with children who may have communication difficulties and their families in ward settings. The training was not intended to focus on children with specific conditions, but all those who experience communication difficulties, whether influenced by learning disability and dysarthria, and/or users of Alternative and Augmentative Communication. It encourages prioritizing communication, cultivating empathy, improving knowledge and developing confidence, and reinforces four key messages around communicating with disabled children: 1) ask the parent/carer for advice on how to communicate best with the child; 2) communicate directly with the child; 3) identify how a child says yes and no; and 4) it is okay not to know how best to communicate with a child and to ask for advice. The training draws upon the theory of planned behaviour,^20^ the construct of self‐efficacy ^21^ and common principles of adult learning, and incorporates behavioural change techniques. The logic model for the intervention can be found in our previous paper.^19^ The intervention is a stand‐alone, peer‐led, 50‐minute training programme, intended to be delivered to mixed groups of ward staff. The training is based around video footage of parent carers discussing real experiences in hospital wards, along with intermittent interactive tasks, discussion, personal reflection and intention planning. Generic and local resources are provided in a handout booklet. The training also aims to raise awareness of communication with disabled children at an organizational level.

The training was previously delivered successfully on several occasions at one hospital.^19^ There was good take‐up with 80 staff attending training across four sessions, and it was well received by participants and the organization. Participant feedback was used to optimize training content and delivery, and all the information required for intervention delivery was documented in a manual with video files. The next step was to evaluate implementing the training in a small number of other hospitals.^22^, ^23^ Considering the context of an intervention, its implementation and mechanisms of impact are important parts of developing and evaluating a complex intervention and can be particularly useful at the feasibility and piloting phase of development in order to optimize intervention design.^22^ Table 1 outlines the aims and objectives of the current study.

**TABLE 1:** Aims and objectives

Aims	Objectives
To investigate the feasibility of implementing the training intervention	To investigate uptake of training by different professions
	To examine the use of the training manual and materials
	To identify the training needs of facilitators
	To identify recruitment strategies used
	To investigate acceptability of the training to ward staff
To review delivery strategies to inform implementation	To identify contextual factors influencing the delivery of training
	To identify approaches used to engage key influencers in the organization to enable training to take place
	To identify contextual and individual influences on participation in training
To determine a framework for evaluating the training	To develop and trial measures to assess staff attitudes, self‐efficacy and self‐reported behaviours before and after training
	To identify contextual and individual influences on use of communication strategies taught
	To identify changes in ward procedures following training

Consideration of implementation was guided by Damschroder's Consolidated Framework for Implementation Research.^24^ This model includes five core domains: the intervention (eg evidence strength and quality), the outer setting (eg recipient needs and resources), the inner setting (eg culture, leadership engagement), individual characteristics and the process (eg plan, evaluate and reflect). We reflected whether constructs relevant to these domains would influence the success of implementation (positively or negatively). This included reflecting on the core and adaptable components of the intervention, and identifying contextual influences on the delivery of training (such as time and clinical pressures, shift patterns, information‐sharing practices, ward culture and relevant policies). It also included reflecting on the approaches used to engage key influencers to enable the training to take place and motivate staff to participate in the training.

## 2 METHODS

### 2.1 Stakeholder involvement

Six parents of disabled children with communication difficulties from the PenCRU Family Faculty collaborated at various times to develop and evaluate the training. Parent carers suggested the topic, helped design the training, recorded experiences for the video content and participated in meetings to reflect on the training delivery. The involvement of parent carers profoundly influenced the content of the training to include real family experiences and deliver messages they feel are important. Paediatricians and nurses were represented, and other ward staff were consulted about the design of the intervention. Disabled students from a local college designed the poster, which acted as an aide memoire on the ward to remind staff of the key messages around communication that are taught in the training.

### 2.2 Setting

We sought at least three hospitals to provide sufficient variability in context to address the research questions. Hospitals in England where there was at least one children's ward were invited to take part via the NHS England Children's Experience of Care Lead. We also registered interest at conferences when presenting on the pilot study.^19^ Interested clinicians made contact with the research team and were asked to identify potential training facilitators and provide contact details for their Research and Development team so that the study researcher(s) could request local sign‐off for the study. Hospitals included in the study therefore consisted of those who volunteered to take part and who had sufficient capacity and local sign‐off to take part.

### 2.3 Participants, recruitment and consent

A senior member of staff at each hospital was identified to oversee the project and was responsible for promoting and facilitating approval for the training to be delivered within the organization. This staff member was also responsible for identifying clinicians to facilitate the training—where more than one clinician was to be involved in facilitating the training, it was recommended that they represent more than one profession. Facilitators were then responsible for advertising, setting up and running the training for their ward/s.

The training was developed for all staff who come into contact with children during, or in preparation for, an inpatient episode. Potential participants consisted of doctors, nurses, allied health professions, receptionists and ancillary staff working on an inpatient or pre‐admission children's ward. Staff attending the training were encouraged to sign up, and read the study information sheet and consent using an online form. The process included a short baseline survey about experience and confidence in communicating with disabled children, their attitudes and reasons for participating, and their knowledge and views about communication support provided within their hospital. Paper versions of these documents were also available. Staff who wanted to attend training but who did not wish to take part in the research were permitted to do so.

### 2.4 Facilitator briefing

Designated facilitators at each hospital were sent a training pack, which included the intervention manual and video files, advertising posters, paper copies of the consent and survey documents, participant handouts and an audio recording device. Prior to delivering training, the facilitators took part in a two‐hour briefing session by teleconference. The facilitator training included a practical run through of the manual and videos, in addition to providing the research background and theoretical underpinning of the study design.

A minimum of six and maximum of 20 participants per training session were recommended to enable group participation and ensure the size of the group was manageable. Hospitals were given the option to target specific professionals or to offer the training more widely, depending on what was possible and also what made most sense in their setting.

### 2.5 Data collection and analysis

Several different approaches and sources of data were used to address the aims and objectives of the research, including the following:

- Training attendance records, completed by facilitators—to capture interest and feasibility of delivery.
- Pre‐ and post‐training anonymous online surveys for staff attending training (Appendix S1)—to capture experience, attitudes and confidence in communicating with disabled children before training and 4‐6 weeks after attending. Survey responses were downloaded and collated in an Excel spreadsheet.
- Training feedback forms (Appendix S2), completed anonymously by staff attending training—to capture staff views on the quality and usefulness of the session and any subsequent behavioural intentions as a result of attending. Feedback forms were collated in an Excel spreadsheet, separated by session and by hospital.
- Audio recording of training delivered—one session to be reviewed to check facilitator fidelity.
- Ad hoc telephone check‐ins with facilitators during the course of the research project—to capture recruitment activities, feedback from sessions, fidelity of delivery and experience of facilitation. Notes from sessions were collated in a document for later analysis.
- Telephone interviews with senior clinicians, facilitators and staff attending training at the end of the study (Appendix S3)—to gather information about the implementation of the training overall, including any barriers or enablers identified, and the impact of training on staff behaviour and organizational policy or practice. Interviews were audio‐recorded and transcribed. Transcripts were reviewed to identify key themes in responses, which were subsequently discussed amongst the research team to confirm accurate interpretation and to reduce bias.

All evaluation measures were reviewed with parent carers. Data collected from the above sources were triangulated where appropriate, and are reported collectively below.

## 3 RESULTS

### 3.1 Participants

Five hospitals including two Children's Hospitals, two District General Hospitals and one University Hospital agreed to take part; one Children's Hospital did not manage to deliver any training during the project period due to time constraints. Hospitals 1 and 2 were involved in the study for 9 months, and Hospitals 3 and 4, for less than 5 months. One senior clinician per hospital volunteered to take part in the study, comprising three Consultant Paediatric Neurologists, one Senior Matron and one Learning Disability Nurse.

Nine training facilitators were recruited (between one and three per hospital); in Hospitals 1 and 3, the senior clinician also acted as one of the (co‐)facilitators. The facilitators differed in their professional roles: three Practice Educators, two Nurses, one Deputy Sister, one trainer for Newly Qualified Nurses, one Learning Disability (LD) Nurse and one Child Disability Clinical Specialist. One or two facilitators delivered the training in any given session. Six facilitators and three senior clinicians took part in telephone interviews at the end of the study, representing Hospitals 1, 3 and 4.

One hundred and nine staff attending training agreed to take part in the research; a further 26 registered for and attended a session but did not provide consent to take part in the research beyond registration (Table 2).

**TABLE 2:** Training sessions and attendees by hospital

Hospital	Type	Training sessions delivered	Staff attending training who consented and completed pre‐training survey (/total attended)	Staff who completed post‐training survey
Hospital 1	District General Hospital	5	33 (/50)	22
Hospital 2	University Hospital with an integrated Children's Hospital	4	37 (/40)	7
Hospital 3	District General Hospital	3	31 (/37)	7
Hospital 4	Children's Hospital	1	8 (/8)	0
Hospital 5	Children's Hospital	0	0	0
Total		13	109 (/135)	36

The participating sites delivered the training to both targeted and mixed groups. Table 3 provides an overview of the different professional roles who attended training, separated by hospital (note this information was gathered at registration, so reports on the full 135 staff who attended). A third of staff had been working in their role for more than 5 years (38%), and just under a third had been in post less than a year (30%).

**TABLE 3:** Overview of different professional roles attending training

	Hospital 1	Hospital 2	Hospital 3	Hospital 4
Advanced Paediatric Nurse Practitioner		4		
Staff Nurse	7	3	5	3
Nursery Nurse	8		1	
Auxiliary Nurse			1	
Specialist Nurse		2		
Student Nurse	3	12	6	1
Nursing Assistant		1	1	
Newly Qualified Nurse				
Learning Disability (LD) Nurse		1		
Sister	4	2		1
Clerical Staff	4			
Play Specialist	2		1	
Health‐care Support Worker/Assistant	4	5	1	
Consultant Paediatrician		1		
Medical Student			10	
Clinical Support Worker				3
Dietician			2	
Community Nurse			2	
Paediatric SHO			1	
Practice Educator		2		
Not provided	1	4		
Total: 109	33	37	31	8

Thirty‐six (39%) staff from three hospitals who attended the training completed the post‐training survey 4‐6 weeks after training took place (see Table 2). These staff covered a range of roles including nurses (Staff, Nursery, Auxiliary, and Student), Health‐care Support Workers, Play Specialists, Medical Students and Clerical Staff. Six staff expressed interest in a telephone interview at the end of the study, but unfortunately, these could not be arranged before the project completed. An email was sent to all consenting participants to ask whether there was any feedback relating to the above they would like to share, but no responses were received.

**FIGURE 1:**
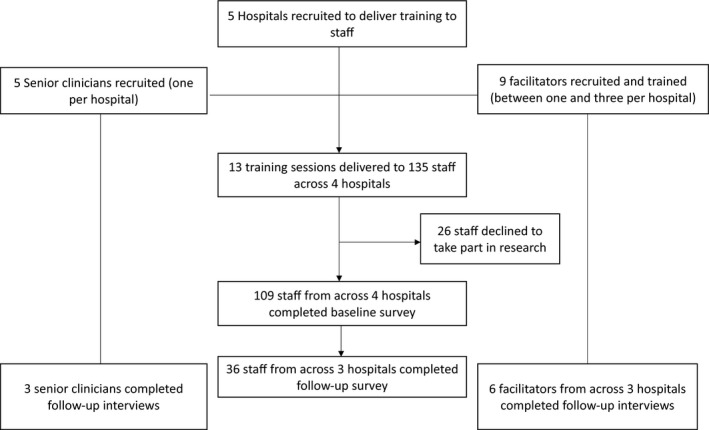
Flow of participants through the study

Figure 1 illustrates the flow and number of participants through the study.

A Template for Intervention Description and Replication (TIDieR) checklist^25^ is included in Appendix S4, which provides an overview of the intervention and the settings in which it has been implemented.

### 3.2 Feasibility of implementing the training and review of delivery strategies

Information gathered from participant surveys and feedback forms, training attendance records, facilitator check‐ins and participant interviews is collated below.

#### 3.2.1 Facilitator preparation

The facilitators who were interviewed reported that they volunteered to take part and indicated their experience of involvement had been positive. The training manual was intended to be self‐explanatory, and facilitators reported finding it easy to use; the telephone briefing session was considered helpful but not essential. Two facilitators who delivered the training session alone said that they would have preferred a co‐facilitator, and another said it would have been useful to experience the training before delivering it.

#### 3.2.2 Advertising and recruiting attendees

Information about recruitment strategies was gathered from Hospitals 1, 3 and 4, who reported using a range of methods to encourage staff to attend training. Training was advertised using the posters provided, and electronically using emails, newsletters and social media across all hospitals. All hospitals made use of pre‐booked training sessions or added the session onto pre‐existing courses, which was considered most effective given the usual time constraints experienced with regard to releasing staff from duty for training. In Hospitals 1 and 3, facilitators also recruited staff from wards on the day. Specific sessions were also arranged for particular groups of staff where a need was identified—for example, in Hospital 1 there was a session for Clerical Staff: ‘Sometimes they are the first people that parents and children will see when they first come through the door’ (Practice Educator). In Hospital 4, they trained staff in A&E, responding to previous requests for training and the acknowledgement that these staff may be less prepared if children did not have a hospital passport to support communication or staff did not know how to use the passport resource appropriately. Facilitators noted that it was difficult to involve certain staff roles, such as doctors, due to shift working practices.

#### 3.2.3 Acceptability of the training

Across all of the hospitals, there was general organizational support for the implementation of this training; for example, ‘We've had a CQC visit… it's really important… they can see the good work… trying to improve what we do. It ticked a lot of boxes’ (Practice Educator, Hospital 1). The main barrier for staff attending was a lack of time or staff capacity to provide cover on the wards, especially, for example, when attempting to engage junior doctors. Where staff were already used to attending training in lunch breaks and where there was support from the ward matron, this enabled staff to attend (Hospital 3).

### 3.3 Evaluating the training: review and impact

#### 3.3.1 Impact on training participants

##### Before training

All 109 staff who took part in the training said they felt some responsibility to interact with disabled children with communication difficulties as part of their role, and over three‐quarters thought this was integral to their work (77%). Almost half reported having daily contact with disabled children (43%), and a further third said that they had contact once or twice per week (34%). Staff confidence in interacting with disabled children prior to attending training varied, with the majority feeling ‘somewhat confident’ (41%) or ‘confident’ (39%). Staff also reported mixed results in terms of how supported they felt by the ward or hospital to interact with disabled children, with the majority feeling ‘supported’ or ‘very supported’ (49%), 41% feeling ‘somewhat supported’ and 10% unsupported. When asked whether they knew where to find local resources to support them to communicate with disabled children, a third did not know (32%) and over half were unsure (52%).

##### Immediately after training

Participants rated the training in the post‐session feedback forms as 9/10 (mean; range = 7‐10, aside from one participant who rated the training as 5/10, commenting that more practical ideas were needed). Participants enjoyed the interactive and discussion elements of the training and hearing about the experiences of parents, and their children made it ‘very real’ and ‘powerful’: ‘The messages to take away are simple but this means they're effective and memorable’ (Student Nurse). Staff overall felt the training was relevant to their role, helpful and easy to understand: ‘It brings home the importance of adapting/altering your communication style for each and every patient’ (Staff Nurse). The majority of participants thought the training would impact on their practice: ‘It made me think about how hard it must be not to be able to talk to others. I will feel more able to ask parents without feeling awkward or shy’ (Health‐Care Support Worker, HCSW). A few participants felt that they already used the techniques covered in the training, but acknowledged that it was a helpful reminder and reinforced current practice.

Facilitators also felt that staff responded well to the training and echoed the importance of hearing from the parents’ perspective: ‘The training highlighted the need for practice to be person‐centred, and not to be afraid to ask’ (LD Nurse). They also found it useful to direct staff to support already available: ‘People didn't realise the extent of the things we have at our disposal’ (Practice Educator). Facilitators also commented that the training made them reflect on their own practice.

##### Four to six weeks after training

Thirty‐six staff returned the follow‐up online survey after 4‐6 weeks, comprising staff from three out of four hospitals where training was delivered; two training sessions delivered by Hospital 2 were delivered too late in the study period to enable collection of follow‐up data (21 staff). Responses from those who completed the survey indicated increased confidence in interacting with children with communication difficulties, with the majority of staff reporting feeling ‘confident’ or ‘very confident’. Staff also agreed the training had helped them to understand the impact of communication on disabled children's experience of care and made them think more about the feelings of children in hospital. Most staff confirmed the training had given them new strategies to use in their interactions with children and their families, and said they had used some of the key messages taught in the training in the last 4‐6 weeks (83%). More staff reported feeling supported by the ward or hospital to interact with children with communication difficulties compared with before the training (56% felt ‘supported’ or ‘very supported’), and over half reported knowing where to find local resources to support communication (53%).

##### Suggested improvements

There were a number of suggested improvements to the training, which participants felt could increase its usefulness and impact, for example adding the child's voice in the videos in addition to the parents’ to the training video; more time to share participants’ own experiences of communication; more information on distraction methods and practical communication tools such as BSL and Makaton; and a longer session to include more information about communicating with children with more complex needs. It was also noted by a number of staff that whilst helpful, the training cannot cover what to do with every child you may come into contact with so difficulties may still arise in practice; ‘Communication is individual’ (Anon). One staff member highlighted the need for good verbal handover of patients and clear documentation and care plans. There was also an acknowledgement that a lack of time can be an issue when communicating with children who may be non‐verbal.

### 3.4 Impact on ward or hospital practices and procedures

At the post‐training follow‐up, a few staff noted small changes to organizational practice guidelines regarding communication with disabled children and their parents, but the majority of staff were unsure whether any changes to practice at ward level had been made (but recognized it might be too soon to tell). In Hospital 1, the facilitators and senior clinician reported that being involved in the research and delivering the training had led to the creation of a working group to take ideas forward. In Hospital 3, the consultant noted that she planned to continue to use the materials and to talk to staff about improving the admissions process so that more information can be gathered about children's needs. There was also recognition from senior clinicians that the hospital needed link nurses in different wards to champion communication. A new learning disability nurse had been recruited in one hospital; the facilitators hoped they would lead continuing work in this area.

All of the hospitals involved in the research interviews intended to deliver the training again and target other groups of staff in the hospital. For example, Hospitals 1 and 3 were keen to include the training in inductions for nurses and junior doctors; Hospital 3 also planned to incorporate it into teaching for students and newly qualified nurses; and Hospital 4 intended to deliver the training at ward training and away days so that it could reach staff from across the hospital.

Facilitators from three hospitals highlighted the need to run through and practise the training before delivery, and ideally to deliver as a pair. One facilitator mentioned that looking up local resources to support communication before the training is important if you are not already familiar with them, as this is helpful for staff to take away. The consultant from Hospital 1 suggested some examples of local resources and pointers about what to look for from the research team would have been useful to support this aspect of training delivery. Because of the time limitations and resource capacity issues experienced by all of the hospitals involved, booking and advertising the training in advance was considered essential. Induction was suggested as a potentially useful point at which to introduce this training to new staff. One consultant reflected that a two‐pronged approach, introducing the training into inductions whilst at the same time trying to reach as many existing staff as possible was the best way to implement it and impact on practice. She also noted that she would like her consultant colleagues to receive the training.

## 4 DISCUSSION

This study provides evidence that local in‐house nurses, practice educators and clinical specialists were able to deliver a focused educational session using our training manual and supporting videos to staff at several different hospitals where children are inpatients. Feedback from staff attending and facilitating training was generally positive, in terms of the programme content and also the impact the training might have on their practice—both at the individual staff member level and the wider ward/organizational level. Whilst it is difficult to measure the real impact of implementing this training on staff behavioural change, the results regarding implementation feasibility and take‐up appear promising. Below, we outline our key findings with regard to our three core aims (Table 1).

### 4.1 Feasibility of training implementation

The study aimed to recruit at least three hospitals to participate and successfully recruited five hospitals even if one was subsequently unable to deliver any training. It also benefited from the mix of general and specialist Children's Hospitals. Hospitals had relatively little time to set up and deliver training to staff, and researchers were also limited on the amount of time available to follow‐up with staff after training, so the number of sessions and staff who benefited from the training was encouraging. The variety of staff who received training was diverse, though lacked representation from particular staff groups including doctors and allied health professionals. Training sessions were often added to existing training or development days.

Feedback from facilitators suggests that the training was received positively by all, and the participant feedback forms suggest that staff took something away with them, even if just a reminder of the importance of communication and an endorsement of their current practice.

Facilitators reported finding the materials easy to use and the training straightforward to deliver. Preparation time, particularly to identify local resources to support communication, was noted as important by most facilitators who were interviewed. Some facilitators suggested incorporating some additional time into the session for discussion as a way of improving the training; it was felt that many staff were keen to share their own experiences of communication, which they viewed as a valuable learning experience to incorporate. This would, however, need to be weighed up against the overall duration of the training considering time was also identified as a barrier to participation in training more generally.

### 4.2 Strategies to inform implementation

Hospitals 1 and 2 participated in the programme for approximately 9 months, but the remaining sites were involved for <5 months. Facilitators were responsible for advertising the training and recruiting staff to attend; posters, emails, social media and newsletters were the main strategies used. The short time available to advertise and deliver training sessions as part of this study was a limitation and inevitably influenced the ability of hospitals to fully implement the training. Indeed, one hospital who was keen to participate failed to arrange a session within the allocated time and therefore was unable to contribute to the research.

As mentioned, the majority of sessions were incorporated or added into pre‐existing time allocated for training and development activities on the ward. As such, many of the sessions were held with groups where participants worked in similar roles (though not all), and where individuals did not volunteer to take part. This means that we have no information about the things that may influence individuals to take part in the training or not. Future research needs to allow more time for hospitals to plan for and recruit participants to enable them to choose how and when best to deliver the training to staff, and to encourage the recruitment of staff who were not involved in this research, such as doctors and allied health professionals.

In accordance with previous studies, the biggest challenge hospitals faced in implementing the training was a lack of time and staff capacity in order to release staff to attend this non‐mandatory training.^26^ In this study, managers and senior staff support enabled these sessions to take place, and facilitators found that adding the sessions to pre‐arranged study days was a useful way to recruit staff. Including the training in induction processes was mentioned by more than one hospital going forward as a way of targeting staff who are otherwise difficult to pin down. This helps to address prior research concerns, suggesting that induction training does not fully prepare health‐care workers for the realities of the ward.^26^ Some facilitators highlighted the time‐consuming nature of the research paperwork required at the start of the session where staff had not signed up online. We suggest that implementing the training will be considerably easier for facilitators and staff when it is not delivered as part of a research study.

### 4.3 Evaluation of training impact

Unfortunately, only 39% of staff who attended the training and completed the baseline survey returned the follow‐up survey, which was sent 4‐6 weeks after the training had taken place. Such a low response rate was unsurprising, but disappointing nonetheless. We cannot therefore make any clear conclusions about the impact of the training on staff. Further, for the training to make a significant impact, it requires commitment to cultural change and leadership in the hospital organization, in staff groups and in individuals over the long term. The limited time provided for such activity within this study is likely insufficient for the training to be delivered to enough staff and create on‐going conversations to impact in a significant and enduring way on hospital culture. Despite this, two hospitals shared that they had plans to create working groups and carry out other more specific actions as a direct result of being involved in the study. All hospitals expressed their intention to continue using the training and resources with staff going forward.

A limitation to this study, and one that was identified by a number of participants, was the absence of disabled children as key stakeholders in the research. Some suggested that including child testimonials in the training videos would further strengthen its impact, and we agree this would be consistent with the fundamental logic model underpinning the training. Whilst local disabled young people were involved in the development of the poster, which accompanied by training (to act as an aid memoire on the ward), only parent carers shared their personal experiences in the training video and represented families on the project steering group.

The results of this research suggest a positive impact of the training session on staff behavioural change, but without further research, neither the magnitude of the effect nor the implications for children and families can be estimated. Conducting such a trial would be complex given staff changes on wards and the varying case mix of children and families.

### 4.4 Future considerations

This training focused on addressing the challenges of communication that may arise on a paediatric ward. Other more comprehensive resources, such as those offered by Disability Matters,^27^ may be more suitable for professionals working with disabled children and their families across different settings. We also recognize the need to evaluate the effectiveness of this training in actually improving children's experience of care as inpatients in hospitals. The premise of the logic model that underpins the training is that increased staff knowledge, skills and confidence will lead to behavioural change, which will in turn improve children's experiences of care.

A key learning point for the researchers involved in the study was the impact of the parent collaborators on all aspects of the design of the programme. The original proposal came from a parent carer, and throughout the design and delivery of the training and research study, we were guided by their experiences in shaping the key messages. We feel that it is important to recognize that although this training was initially developed with disabled children in mind, the principles and key messages about good practice when communicating with children and families are applicable to all children who visit a paediatric ward, and indeed could be extended to communicating with vulnerable adults and adults with communication difficulties.

Furthermore, it is useful to note that whilst the training was developed for face‐to‐face delivery, given that the video clips and subsequent discussion points form the main part of the training, there is the potential for this session to be delivered virtually. This is important to note given that current working practices are moving into an increasingly virtual world, and also opens up the possibility of the training being delivered to practitioners who otherwise would be unable to attend.

### 4.5 Conclusions

Despite national and international policies calling for action to improve communication and inpatient hospital experiences for disabled children, these inequalities persist. Under the day‐to‐day pressures of providing health care, this agenda can become lost. This study demonstrates a desire and need for training in this area, supporting the delivery of a low‐cost, fixed‐time, minimal‐resource training package to raise awareness about good practice when communicating with children and their families at the organizational level. The training package can be delivered in diverse hospital settings, and staff both delivering and receiving the training appear to find it useful.

## CONFLICT OF INTEREST

None to disclose.

## AUTHORS' CONTRIBUTIONS

CM, RG & SL designed the overall project and consulted with parent carers, supported by HH. KW carried out the research and maintained relationships with parent carers and clinicians throughout. All authors contributed to the drafting of the manuscript.

## Supporting information

Appenidx S1

Appendix S2

Appendix S3

Appendix S4

## Data Availability

The data that support the findings of this study are available from the corresponding author upon reasonable request.
