# Supplementary material for: Implementation of training to improve communication with disabled children on the ward: A feasibility study
Source: Health Expect. 2021 May 28;24(4):1433–42. doi: 10.1111/hex.13283 (PMC8369114; doi:10.1111/hex.13283)
Supplement: Supplementary file 1 — Appenidx S1 [file HEX-24-1433-s004.docx]

**Appendix S1. Pre- and post-training online surveys for training participants**

**Hospital Communications Training – Participant registration**

**Communicating with Disabled Children in Hospital Training - Online registration**

Top of Form

Thank you for completing this registration form for the training: Communicating with Disabled Children in Hospitals

The training was co-developed by health professionals and families of disabled children in Exeter, and Exeter Univiersity are now researching delivery of the training in other UK hospitals to see if it works well in other settings.

The answers you provide below, along with your feedback after the training, will help us to identify if the training can be delivered beyond Exeter, and what, if any, modifications need to be made to make it as useful as possible.

We will not report your name in any analyses or share your personal details beyond the research team.

Top of Form

**About you**

Top of Form

1 Which hospital do you work at? Top of Form

a. What is the name of the ward you work on?

Top of Form

2 What is the date of the training session are you signing up for?

Top of Form

3 What is your name? (Your name will only be used to link your responses before and after training and to confirm attendance. It will be removed from your answers for analysis and reporting.)

Top of Form

4 What is your job title?

Top of Form

a What is your staff grade?

Top of Form

b How long have you been working in this role?

Top of Form

- Less than a year
- 1-2 years
- 3-5 years
- More than 5 years

5 What is your email address? (This will be used by the research team to send the training evaluation and to provide updates and a report at the end of the study.)

Top of Form

**Pre-training questions**

Top of Form

6 How did you hear about this training opportunity?

Top of Form

7 Do you have any special requirements for participating in the training, such as physical, visual or language requirements? (1 not at all confident – 5 very confident)

Top of Form

Please complete the following questions bearing in mind the disabled children who are on your ward(s), such as children with learning disabilities or children who use communication aids.

Top of Form

8 In your professional role, how often do you come into contact with disabled children?

Top of Form

- Every day
- Once or twice a week
- Once or twice a month
- Less than once a month

9 Do you feel a personal responsibility to interact with disabled children as part of your role? Circle on a scale 1 to 5.

- 1 - Not at all, it's not my role
- 2
- 3
- 4
- 5 - Very much, it's integral to my work

Top of Form

10 How confident do you feel interacting with disabled children?

- 1 - Not at all confident
- 2
- 3
- 4
- 5 - Very confident

Top of Form

Bottom of Form

11 Do you feel supported by the ward or hospital to interact with disabled children?

- 1 - Not at all supported
- 2
- 3
- 4
- 5 - Very supportedTop of Form

12 Do you know where to find local resources to support communication with disabled children?

Top of Form

- Yes
- No
- Not sure

a If yes, where?

**Top of Form**

**Consent to participate in the research**

Top of Form

Since this training is being provided as part of a research project, you will need to consent to taking part in the follow-up online survey. Please read each statement below and confirm that you agree to take part in this research project.

 Top of Form

13 I have read the information sheet emailed to me and understand what the research is about. I understand that I am free to ask questions and request further information at any stage.

Top of Form

- Yes
- No

14 I understand that my participation in the project is entirely voluntary.

Top of Form

- Yes
- No

15 I understand that I am free to withdraw from the project at any time without any disadvantage. Top of Form

- Yes
- No

16 I understand that the training will be audio recorded and listened to by a researcher.

Top of Form

- Yes
- No

17 I understand that the data, including audio data, will be retained in secure storage.

Top of Form

- Yes
- No

18 I understand that the results of the project may be published but my anonymity will be preserved.

Top of Form

- Yes
- No

19 I understand that my responses in the feedback form will be anonymous. Feedback forms will be made available to facilitators to review.

Top of Form

Bottom of Form

- Yes
- No

20 I understand that my responses to the survey will only be seen by the research team. The trainers will only see survey results once they are published.

Top of Form

- Yes
- No

21 I agree to take part in this project.

Top of Form

- Yes
- No

**Thank you!**

Top of Form

Many thanks for taking the time to complete this registration. We hope that you enjoy the training and find it useful.

We will email you a link after you have attended the training so that you can complete an evaluation. It is essential for us to get your feedback and know how the training is working and if it needs to be changed or improved.

**This project has been reviewed and approved by the University of Exeter Medical School Research Ethics Committee (UEMS REC REFERENCE NUMBER: Nov17/B/134).**

**Communicating with Disabled Children in Hospitals - Post-Training Questionnaire**

**About you**

Top of Form

Bottom of Form

You have been invited to complete this questionnaire because recently you attended the following training: Communicating with Disabled Children in Hospitals

The training was co-developed by health professionals and families of disabled children in Exeter, and Exeter Univiersity are now researching delivery of the training in other UK hospitals to see if it works well in other settings.

The answers you provide below will help us to identify if the training can be delivered beyond Exeter, and what, if any, modifications need to be made to make it as useful as possible.

We will not report your name in any analyses or share your personal details beyond the research team.

Top of Form

1 Which hospital do you work at?

Top of Form

a What is the name of the ward you work on?

Top of Form

2 What is the date of the training session you attended?

Top of Form

3 What is your name? (Your name will only be used to link your responses before and after training and to confirm attendance. It will be removed from your answers for analysis and reporting.)

Top of Form

4What is your email address? (This will be used by the research team to send the training evaluation and to provide updates and a report at the end of the study.)

Top of Form

**Working with children with communication difficulties**

Top of Form

Thinking about children with communication difficulties who are on your ward(s), such as children with learning disabilities or children who use communication aids.

Top of Form

5 Do you feel a personal responsibility to interact with children with communication difficulties as part of your role?

Top of Form

- 1. Not at all, it's not my role
- 2. Mostly no, it's not really my role
- 3. Neither part of my role or not
- 4. Slightly, it's part of my role
- 5. Very much, it's integral to my role

6 How confident do you feel interacting with children with communication difficulties?

- 1. Not at all confident
- 2. Not very confident
- 3. Somewhat confident
- 4. Confident
- 5. Very confident

Top of Form

7 Do you feel supported by the ward or hospital to interact with children with communication difficulties?

- 1. Not at all supported
- 2. Not very supported
- 3. Somewhat supported
- 4. Supported
- 5. Very supported

Top of Form

8 Do you know where to find local resources to support communication with these children?

Top of Form

- Yes
- No

9 In your recent interactions with children with communication difficulties, did you use any of the 4 key messages taught in the training?

Top of Form

- Yes
- No

a If yes, please tick one or more strategies that you used

Top of Form

- Asking parents how to go about things before procedures
- Talking directly to the child, not just the parent
- Identifying how a child says yes/no
- Acknowledging that it can be stressful or embarrassing to not know how to communicate with a child

**About the training**

Top of Form

We have been trialling the training to see how useful it is to ward staff and to make improvements. Please tell us what you think about the training.

Top of Form

10How much has the training helped you in your interactions with children with communication difficulties? For each item, mark your answer on the scale (where 1 is not at all and 5 is very much).

Top of Form

|  | 1. Not at all | 2. Not very much | 3. Somewhat | 4. Quite a bit | 5. Very much |
| --- | --- | --- | --- | --- | --- |
| It has helped me to understand the impact of communication on disabled children's experience of care. | Checkbox | Checkbox | Checkbox | Checkbox | Checkbox |
| It has made me think more about the feelings of children in hospital. | Checkbox | Checkbox | Checkbox | Checkbox | Checkbox |
| It has given me strategies that I can use in my interactions with children and their families. | Checkbox | Checkbox | Checkbox | Checkbox | Checkbox |

11 Are there any other ways in which the training has helped you?

Top of Form

12 When interacting with children with communication difficulties, are there things that you still find difficult or that the training hasn’t helped with?

Top of Form

13 Have there been any changes to routine practices or procedures on the ward as a result of the training, such as changes to information sharing procedures?

Top of Form

- Yes
- No
- No sure

a Please specify any changes

Top of Form

14 Do you have any suggestions for improving the training designed to support staff’s interactions with children who have communication difficulties?

Top of Form

**Research next steps**

Top of Form

We wish to interview up to 24 training participants to find out more about participants’ experiences of training and of using the training in practice.  If you are selected to participate, we will send you further information about the interview by email so you can decide whether or not to take part. We will be selecting participants from a range of professions and locations.

Top of Form

15 Are you interested in participating in a short telephone interview (10-15 minutes) about your experiences of the training?

Top of Form

- Yes
- No

**Thank you!**

Top of Form

Thank you very much for completing this survey.

Top of Form
