# Supplementary material for: Implementation of training to improve communication with disabled children on the ward: A feasibility study
Source: Health Expect. 2021 May 28;24(4):1433–42. doi: 10.1111/hex.13283 (PMC8369114; doi:10.1111/hex.13283)
Supplement: Supplementary file 2 — Appendix S2 [file HEX-24-1433-s001.docx]

**Appendix S2. Anonymous post-training participant feedback form**

**Improving Inpatient Experiences for Disabled Children**

**Participant Feedback Form**

We would be grateful for your feedback on the training session that you received today. The feedback is anonymous.

1. What is your professional role?
2. Please score out of 10 for training Content, Organisation, Delivery, Relevance and Overall

| **Area** | **Score out of 10** |
| --- | --- |
| **C**ontent |  |
| **O**rganisation |  |
| **D**elivery |  |
| **R**elevance |  |
| **O**verall |  |

1. Please give us your feedback on the training session. We welcome critical feedback so that we can make improvements, as well as feedback on what was good about the training
2. Do you think that attending this session today will change your practice? If yes, how?
