# Supplementary material for: Implementation of training to improve communication with disabled children on the ward: A feasibility study
Source: Health Expect. 2021 May 28;24(4):1433–42. doi: 10.1111/hex.13283 (PMC8369114; doi:10.1111/hex.13283)
Supplement: Supplementary file 3 — Appendix S3 [file HEX-24-1433-s003.docx]

**Appendix S3. End of study interview schedules**

**Telephone interview with facilitators at each site:**

This is a semi-structured interview. Questions may vary depending on what is known already about how the training delivery has gone. The aim of the interview is to identify any changes needed to the manual and materials and to identify barriers to delivery of the training and any impacts of the training they have observed on ward practice. For example:

Facilitator name:

Job title:

Ward:

Staff grade:

Length of time working in this role:

Ward where training took place – characteristics:

1. How did you find out about the project?
2. How did you become a facilitator for the training?

*Volunteered? Nominated?*

1. What was your experience of being a facilitator?
   1. Was it a positive experience? Why/why not?
   2. Was there anything negative about it, i.e was it stressful?
   3. How did you find the time commitment of being a facilitator? Was it feasible?
   4. Did it make you reflect on your own practice?
2. You participated in the online/over the phone training for facilitators – how useful did you find this?

*Is some sort of training like this needed, or is it feasible without?*

*Would you have liked any further support as a facilitator?*

a. What else did you do to prepare to deliver the training?

b. How did you prepare the local resources section? What did you find out and what did you share in the training?

1. Do you have any suggestions for changes to the training manual or materials?
   1. Was the manual clear and easy to follow? How/why?
   2. Were the supporting materials in the training pack useful? Anything particularly useful? Did you use all of the materials provided?
   3. Was there anything missing that would have been useful to include?
2. What did you do to advertise the training in your hospital?
   1. Were there any strategies that you found more/less successful at encouraging staff to sign up?
   2. Did you target any particular groups? Who? How?
   3. Did you notice any change in staff sign-up after the initial session or two? I.e. were people more likely to sign up after they had heard about it from others?
3. Have you felt supported by your organisation to deliver the training?
   1. What are the organisational and ward barriers and facilitators to delivery of the training?
   2. Is there a training culture in your hospital/ward?
   3. How easy did you find it for staff to find time to attend?
      1. Did you need to speak to managers directly for permissions?
      2. Was everyone on board? I.e. service/ward managers, clinical managers, line managers etc.
4. How do you feel staff have responded to the training?

*Did you notice any differences between professional groups?*

1. Has the training highlighted any needs for changes in ward practice to support communication with disabled children and their families?
   1. Have you observed, or heard about any changes in ward practice to date?
   2. Are there any future plans to make changes?
2. Would you suggest doing anything differently if you were going to implement the training in another hospital?
3. Anything else you’d like to tell us?

**Telephone interview with senior staff member at each site:**

This is a semi-structured interview. Questions may vary depending on what is known already about how the training delivery has gone. The aim of the interview is to identify contextual influences in the delivery of training.

Champion name:

Job title:

Ward:

Staff grade:

Length of time working in this role:

1. How did you find out about the project?
2. How did you become champion for the project?
3. How did you choose the facilitators?
4. What are the characteristics of hospital? (could ask for another person to provide this information if preferable)
   1. Type of hospital? I.e. University, or District General Hosp, or Children’s etc.
   2. Number of beds for children (capacity)?
   3. Are there any learning disability-specific roles in place?
   4. Are there particular wards where children might be have more complex needs/communication problems e.g. neurological ward/rehab?
5. Was the training focused on a particular ward? If yes, what re the ward characteristics?
6. How were you involved in the training?
7. If you participated in the online/over the phone training for facilitators – how useful did you find this?

*Is some sort of training like this needed, or is it feasible without?*

*Would you have liked any further support for facilitators?*

1. Did you help to advertise the training in your hospital?
   1. Were there any strategies that you found more/less successful at encouraging staff to sign up?
   2. Did you target any particular groups? Who? How?
   3. Did you notice any change in staff sign-up after the initial session or two? I.e. were people more likely to sign up after they had heard about it from others?
2. Have you felt supported by your organisation to deliver the training?
3. What are the organisational and ward barriers and facilitators to delivery of the training?
4. Is there a training culture in your hospital/ward?
5. How easy did you find it for staff to find time to attend?
   - 1. Did you need to speak to managers directly for permissions?
     2. Was everyone on board? I.e. service/ward managers, clinical managers, line managers etc.
6. How do you feel staff have responded to the training?

*Did you notice any differences between professional groups?*

1. Overall, how do you think the training has been received in your hospital?
2. Has the training highlighted any needs for changes in ward practice to support communication with disabled children and their families?
3. Have you observed, or heard about any changes in ward practice to date?
4. Are there any future plans to make changes?
5. Would you suggest doing anything differently if you were going to implement the training in another hospital?
6. Anything else you’d like to tell us?
