# Supplementary material for: Implementation of training to improve communication with disabled children on the ward: A feasibility study
Source: Health Expect. 2021 May 28;24(4):1433–42. doi: 10.1111/hex.13283 (PMC8369114; doi:10.1111/hex.13283)
Supplement: Supplementary file 4 — Appendix S4 [file HEX-24-1433-s002.docx]

**Appendix S4. Template for Intervention Description and Replication (TIDieR)**

| **Name** | Training to improve communication with disabled children on the ward |
| --- | --- |
| **Why** | - Parents of disabled children report poorer inpatient experiences when they stay in hospital. - Communication is a key determinant of inpatient experience. - Ward staff report finding communicating with disabled children challenging. - NHS policy prioritises enhancing patient experience.   This short, minimal-resource training was developed with clinicians and parent carers. It aims to increase the knowledge and confidence of hospital staff in how best to communicate with disabled children and their families whilst they are inpatients in hospital. |
| **What** | A 50-minute training session led by in-house hospital staff, aiming to:   - Convey 4 key practices to improve inpatient experience - Cultivate empathy - Develop confidence - Identify local resources - Raise awareness within the organisation   The training draws on the theory of planned behaviour, the construct of self-efficacy, and common principles of adult learning, and incorporates behaviour change techniques.  Training comprises a stand-alone 50-minute video including:   - An ice-breaker activity - Footage of parent carers talking about real life experiences in hospital wards - Interactive tasks and discussion points - Personal reflection - Intention planning - Sharing of local resources - A handout including research papers relevant to the training and a poster of the 4 key messages taught, which was designed by disabled students.   Facilitators receive a manual, including tips, scripts and discussion points to support delivery, as well as background information about the intervention and relevant research papers.  Resources can be requested from the authors. |
| **Who** | Training was designed to be delivered by in-house hospital staff, and no intervention training is required. There is a facilitator manual to support staff delivery of the training.  The trainers should have enthusiasm for this topic and knowledge of local resources available or being planned to improve children’s experiences as inpatients. We have found that the training works best with one person hosting the event, and others assisting to deliver particular activities or provide information about the local resources available to support communication. At least one of the trainers should be a familiar peer of participants. Ideally the trainers will include both a nurse and a doctor, or other staff member, so that more than one professional role are represented among the trainers.  Trainers should be able to engage participants, present material in a way that is relevant to their situation and encourage continuation of the learning processes. Skills required include group work facilitation, keeping to time and building confidence in others. |
| **How** | The intervention is designed to be delivered face-to-face in a group, with one or two facilitators. The training is intended for all professionals who come into contact with children on the ward within a hospital setting, including porters, catering staff, cleaners, receptionists, as well as doctors, nurses and allied health professionals. This is because responsibility for communication does not rest upon one professional group, and that improvements in communication can be made across all routine interactions on a ward.  It has been successfully delivered to a variety of staff groups of between 6 and 20 per session. |
| **Where** | Training has been successfully delivered to staff working on Children’s Wards in five English hospitals;   - One Children’s Hospital - Three District General Hospitals - One University Hospital |
| **When and how much** | In the pilot study in 2016-17, the training was delivered in one hospital, with 80 staff attending across 4 sessions.  In the feasibility study in 2018, the training was delivered in four hospitals:   - Hospital 1 - training to 50 staff over 5 sessions (over 9 months) - Hospital 2 - training to 40 staff over 4 sessions (over 9 months) - Hospital 3 - training to 37 staff over 3 sessions (over 5 months) - Hospital 4 - training to 8 staff over one session (over 5 months) |
| **Tailoring** | There is no tailoring. The training is developed to be suitable for all professionals working in hospitals where they may come into contact with disabled children. |
| **Modifications** | The training materials were refined slightly following the pilot study to provide further guidance for facilitators in the manual. |
| **How well** | Researchers provided an initial online training session for facilitators to go over the training. They also conducted regular check-ins with facilitators before and after training sessions were delivered to gather feedback on acceptability, feasibility and fidelity of delivery. Reports suggested that the training was delivered by facilitators as planned and with little difficulty.  Additional pans to review recordings of training sessions unfortunately did not materialise. |
